# A multivariate model of IVIM-DWI in the preoperative diagnosis of tumor budding grade in rectal cancer

**DOI:** 10.3389/fonc.2025.1543607

**Published:** 2025-06-09

**Authors:** Junyi Fu, Chenglong Zhao, Guangying Zheng, Aiyin Li, Gesheng Song

**Affiliations:** ^1^ Department of Radiology, The First Affiliated Hospital of Shandong First Medical University & Shandong Provincial Qianfoshan Hospital, Jinan, China; ^2^ Department of Pathology, The First Affiliated Hospital of Shandong First Medical University & Shandong Provincial Qianfoshan Hospital, Jinan, China; ^3^ Shandong First Medical University and Shandong Academy of Medical Sciences, Jinan, Shandong, China

**Keywords:** rectal cancer, tumor budding, magnetic resonance imaging, diffusion weighted imaging, clinical pathology

## Abstract

**Objective:**

The aim of this study was to explore the application value of intravoxel incoherent motion diffusion-weighted imaging (IVIM-DWI) in the preoperative evaluation of the tumor budding (TB) grade in patients with rectal cancer (RC).

**Methods:**

Patients with RC who underwent rectum resection from January 2018 to October 2023 were collected retrospectively. All patients underwent magnetic resonance (MR) examination, including collection of IVIM sequences, within 1 week before surgery. Among them, 17 low-grade and 13 intermediate-grade budding cases were classified into the low-intermediate-grade group, while 30 high-grade budding cases were classified as high grade. After processing the IVIM sequences, the apparent diffusion coefficient (ADC) from the mono-exponential (ME) model; *D*, D*, and *f* from the bi-exponential (BE) model; and the DDC and α from the stretching (SE) model were obtained. Clinical factors, including age, gender, and CEA levels, and imaging factors, including location, mriT, and N stage, were collected. Differences between the low-intermediate- and high-grade groups were compared. The diagnostic efficiency was evaluated from the ROC curve, AUC, sensitivity, and specificity. Significance was set at *p* < 0.05.

**Results:**

A total of 60 patients with RC were enrolled. Significant differences between low-intermediate and high grade were found in *f* value (*p* = 0.001), DDC value (*p* < 0.001), age (*p* = 0.002), location (*p* = 0.047), and mesorectal fascia (MRF) (*p* = 0.01). Multivariate binary logistic regression analysis identified *f*, DDC, MRF, and age as independent risk factors (*p* < 0.05). The AUC of the combined model (*f*, DDC, MRF, and age) was 0.920 (95% CI, 0.820–0.974), with a sensitivity and specificity of 1.000 and 0.767, respectively.

**Conclusion:**

The combined model constructed using multiple IVIM mathematical model parameters and clinical and imaging factors could be used as a noninvasive tool to facilitate the preoperative auxiliary diagnosis of TB in RC.

## 1 Background

Rectal cancer (RC) has arisen as one of the most common tumors of the digestive tract (1), with an increasing incidence and high mortality (2). Most treatment options refer to the TNM tumor staging system, but the treatment effect is significantly different (3, 4). Tumor budding (TB) is a histological feature of tumor cells, defined as the detection of a single cell or a cell cluster of ≤4 cells at the front edge of an invasive cancerous tumor by hematoxylin and eosin (H&E) staining (5). TB can be divided into three grades: 0–4 tumor buds, low-grade budding; 5–9, intermediate-grade budding; and ≥10, high-grade budding. Studies have shown that TB was related to the histological grade, nerve invasion, lymph node metastasis, and tumor stage of colorectal cancer (6), and is an independent adverse prognostic factor for colorectal cancer (5, 7–11). Some studies have shown that TB is an independent risk factor for the specific survival of colorectal cancer, with greater significance and a higher relative risk ratio than other clinicopathological parameters (12). Because of its unique prognostic value, it can further guide the treatment strategy and adjuvant treatment (13, 14). However, the evaluation of budding grade is only confirmed in postoperative pathology. Predicting the TB grade from perspective imaging before treatment is of great significance for the formulation of a clinical treatment plan and may affect the prognosis and survival rate of patients to a certain extent.

As a non-invasive imaging technology, magnetic resonance imaging (MRI) has been widely applied to evaluate RC preoperatively and to predict prognosis (15, 16). Le Bihan et al. (17) first proposed diffusion-weighted imaging (DWI) based on intravoxel incoherent motion (IVIM) in 1986. IVIM-DWI can distinguish between the diffusion effect of water molecules in biological tissues and the microcirculation perfusion effect through the measurement of multiple *b* values. Through different mathematical model processing, including mono-exponential (ME), bi-exponential (BE), and stretching (SE) modeling, ADC, *D*, D*, *f*, DDC, and α values can be obtained. ADC and *D* reflect the diffusion information represented by the fast diffusion component; D* and *f* reflect the perfusion information represented by the slow diffusion component in the voxel; DDC reflects the average intravoxel diffusion rate; and α reflects the intravoxel distribution of apparent diffusivity (18).

IVIM has also been shown to be of great significance in the evaluation of certain pathological features of RC. For example, Hong et al. (19) pointed out that IVIM can be used as a preoperative auxiliary diagnostic tool to predict the tumor deposition of RC. Some researchers (20) have also found that IVIM-DWI-derived parameters of patients with RC are related to tumor grade and tumor stage and can diagnose whether extramural venous invasion (EMVI) is positive or not. Thus far, there has been no study on the evaluation of RC TB grade using DWI-IVIM. Therefore, this study used various mathematical models to analyze data obtained by DWI-IVIM to diagnose RC TB.

## 2 Methods

### 2.1 Patient population

**Figure 1:**
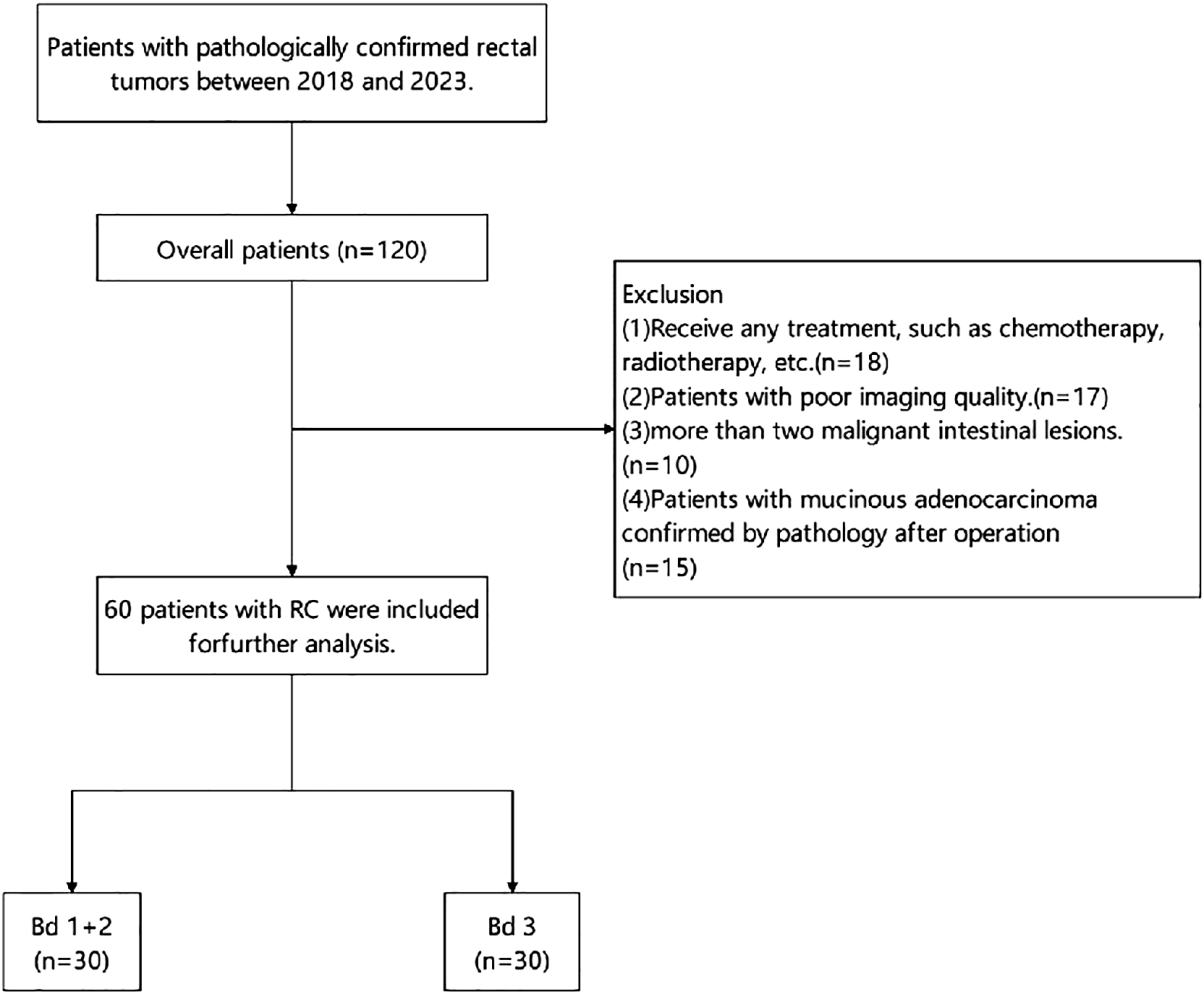
Flowchart of patient enrollment.

This study was approved by the First Affiliated Hospital of Shandong First Medical University & Shandong Provincial Qianfoshan Hospital institutional review board (IRB:S974). The study design involved the retrospective analysis of 120 patients with pathologically confirmed RC who underwent pelvic MR, including high-resolution T2-weighted imaging and multi-*b* value DWI from 2018 to 2023. The exclusion criteria were as follows: (a) any prior treatment, such as chemotherapy and radiotherapy; (b) patients with poor imaging quality; (c) multiple rectal malignant lesions; and (d) pathological confirmation as mucinous adenocarcinoma post-operatively. Finally, a total of 60 eligible patients were included in the study and divided into two groups: the low-intermediate-grade and high-grade TB groups. **Figure 1** presents the grouping flowchart of the research queue. The patient’s age, gender, pathological stage, and some laboratory test results (CA199/CA125) were included in the study as clinical factors from the clinical and laboratory information.

### 2.2 MRI acquisition

All patients who took part in this study underwent 3.0 T MR (Discovery 750; GE Healthcare) using an eight-channel phased array body coil in the supine position 2 weeks prior to surgery. Participants followed a liquid diet for 2 1/2 days before the examination, and an enema was conducted to empty the colon of feces prior to the examination. On the day of the examination, 10 mg of anisodamine (654-2) was intramuscularly injected 30 min pre-imaging to reduce the interference of intestinal peristalsis artifacts on the examination. Before scanning, the patient’s rectum was filled with ultrasound projection gel to fully expand the rectum, and the scanning range included all pelvic cavities. The imaging protocol included sagittal T2WI, coronal T2WI, oblique HR-T2WI (high-resolution T2-weighted imaging) images (perpendicular to the long axis of the intestinal tube where the tumor was located), and axial DWI (including 11 *b* values, 0, 20, 50, 100, 150, 200, 400, 600, 800, 1,000, and 1,500 s/mm^2^). The scanning sequence and parameters are listed in **Table 1**.

**Table 1:** Scanning sequence and parameters.

Sequence	TE (ms)	TR (ms)	FOV (cm)	Thickness (mm)	Gap (mm)	Matrix	Acquisition time (s)
Sagittal T2WI	85	8,137	27	4	0.4	352×352	199
Oblique axis HR-T2WI	102	5,868	18	3	0	320×288	286
Coronal T2WI	90	7,382	32	4	0.4	480×480	231
Axial IVIM-DWI	63.7	5,000	32	4	0.5	96×128	535

### 2.3 MRI image postprocessing solution

**Figure 2:**
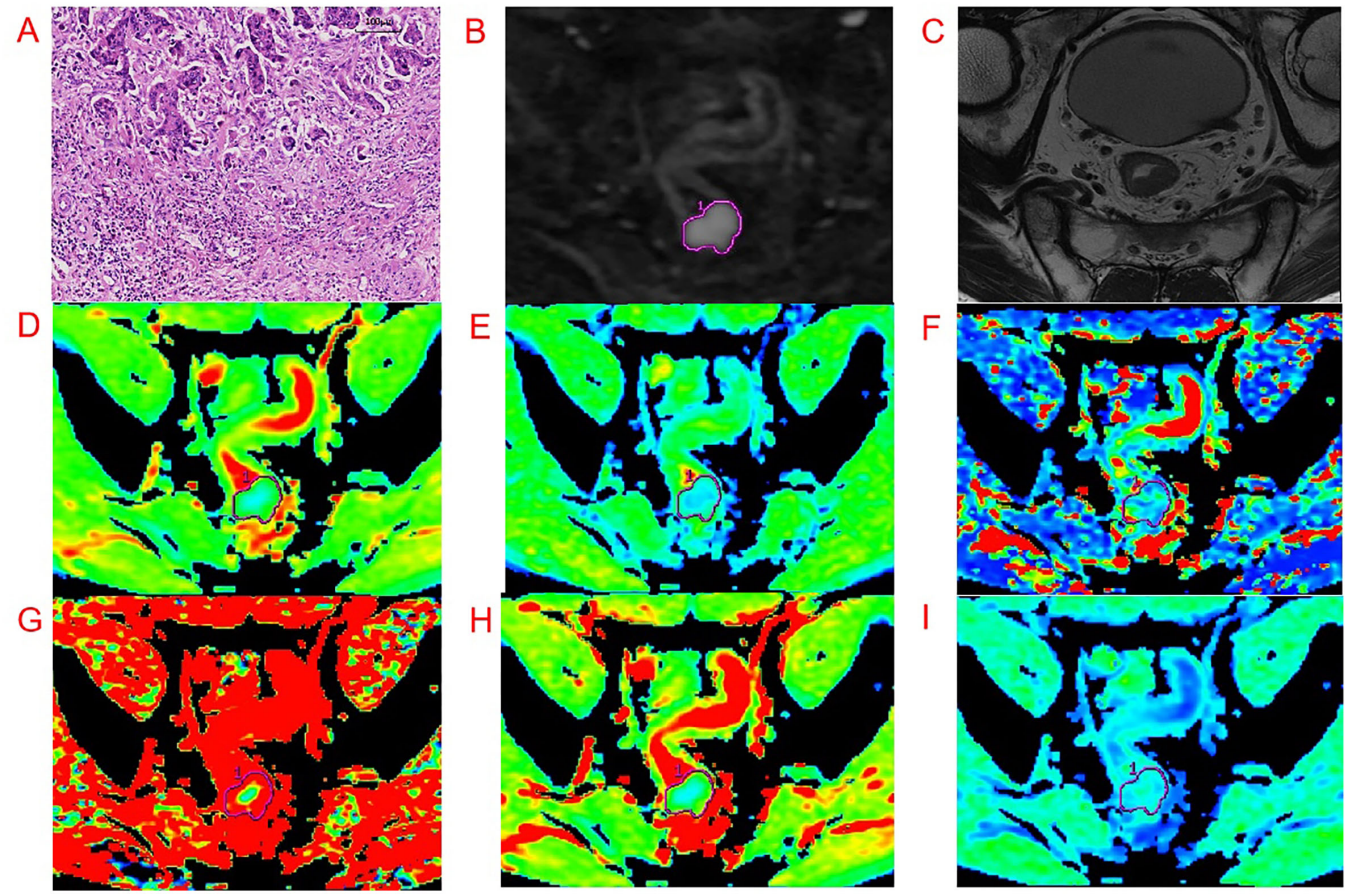
Representative images from an example 54-year-old male patient with RC with pathologically proven with Bd 3. Regions of interest were manually drawn along the border of the tumor on DWI, with a *b*-value of 1,000 s/mm^2^ and directly co-localized on all parametric maps. **(A-I)** are pathological imaging, DWI, oblique axial T2WI, ADC, *D*, D*, *f*, DDC, and α map, respectively.

All IVIM sequence images were transferred to an AW4.6 GE medical systems workstation, and image processing was performed using the functool MADC software. Two radiologists with experience in RC diagnosis (reader 1 and 2) independently drew the regions of interest (ROIs). If one of the drawn ROI was found to have low consistency, it was re-measured by the senior radiologist. ROI delineations were manually drawn on the DWI sequence, targeting a *b* value of 1,000 s/mm^2^ for the ROI placed on the solid part of the tumor body, and locally protrude to the front edge of the tumor infiltrating side. We attempted to avoid any regions of necrosis, hemorrhage, etc., and selected the largest layer of the lesion as the central layer, thereafter continuously drawing three planes to obtain the relevant mathematical parameters. The average value was obtained as the final data. A representative image is shown in **Figure 2**.

### 2.4 Tumor evaluation and image feature acquisition

The MRI reports of each patient were evaluated by two radiologists (Readers 1 and 2 with 15 and 6 years of experience, respectively). The TN stage on MRI was evaluated based on the American Joint Committee on Cancer (AJCC) guidelines. A positive diagnosis of lymph metastatic nodes was made if the lymph nodes had a short diameter of >5 mm and included one or more features of uneven signal, irregular shape, and unclear boundary. The primary site was divided into three grades: low, medium, and high, according to the distance between the primary site and the anal edge. The mrEMVI score and MRF/peritoneal status were evaluated on oblique axis HR-T2WI. EMVI evaluation was performed using a modified version of the five-point scoring system outlined by Smith et al. (21). Positive mesorectal fascia (MRF+) was defined as a distance of ≤1 mm between the primary lesion of RC, metastatic lymph nodes in the mesorectal region, and EMVI and MRF. When the diagnosis results of the two radiologists differed, a radiologist with 20 years of experience in the diagnosis of RC made the final diagnosis.

### 2.5 Pathology evaluation

All tissue sections underwent H&E staining. Histopathology results included tumor TN staging, histological grade, presence of perineural invasion, presence of lymph-vascular invasion (LVI), tumor deposits, and descriptions of the circumferential resection margins.

H&E-stained sections were used to determine the area with the densest budding under low magnification, and TB counts were performed on the selected area under high magnification. The TB grade was expressed by the Bd based on the International Tumor Budding Consensus Conference standard (5), as follows: Bd 1 (0–4 buds), Bd 2 (5–9 buds), and Bd 3 (≥10 buds), representing low-, intermediate-, and high-grade TB, respectively. Two pathologists (both with >8 years of experience in pathology) independently completed all pathological diagnoses and performed statistics and scoring. Patients were divided into two groups: low-intermediate (Bd 1+ Bd 2) and high grade (Bd 3) for analysis.

### 2.6 Statistical analysis

All statistical analyses were performed in SPSS26.0 (IBM, Armonk, NY, USA) and MedCalc 20.0 (MedCalc, Mariakerke, Belgium). For continuous variables that conformed to the normal distribution and met the homogeneity of variance, the independent-sample *t*-test was used for comparison. Conversely, for data that did not conform to the normal distribution or homogeneity of variance, the Mann–Whitney *U* test was applied. Finally, for categorical variables, the chi square test or Fisher exact test was used to compare statistical differences. The diagnostic reliability of the two radiologists was evaluated according to the intraclass correlation coefficient (ICC), and a correlation model was established according to the indicators of *p* < 0.05 in each group. At the same time, binary logistic regression analysis was applied to obtain the independent risk factors of TB. Furthermore, receiver operating characteristic (ROC) curves were drawn to identify the diagnostic performance, while the Delong test was applied to compare the differences in the areas under the curves (AUCs). *p* < 0.05 was considered statistically significant.

## 3 Results

### 3.1 Patients’ characteristics

The 60 patients included in the study were divided into two groups. The low-intermediate-grade group included 19 male and 11 female patients and the high-grade group included 22 male and 8 female patients. There were no significant differences in gender, CEA, CA199, MRI-t, MRI-n, or EMVI stages between the two groups (*p* > 0.5). **Table 2** shows the details of clinical and imaging characteristics of the patients in both groups.

**Table 2:** Relevant clinical, pathological, and radiological details of the enrolled patients.

Characteristics	TB grade		*p*
	Low-intermediate (*n* = 30)	High (*n* = 30)	
Age (years), mean ± SD	66.53 ± 1.95	58.60 ± 1.520	0.002
Gender, *n* (%)			0.405
Male	19 (63.3)	22 (73.3)	
Female	11 (36.7)	8 (26.7)	
CEA (ng/mL), *n* (%)			0.190
<5	20 (66.7)	15 (50.0)	
≥5	10 (33.3)	15 (50.0)	
CA199 (U/mL), *n* (%)			1.000
<37	27 (90.0)	27 (90.0)	
≥37	3 (10.0)	3 (10.0)	
Tumor location, *n* (%)			0.047
Upper	7 (23.3)	14 (46.7)	
Middle	13 (43.3)	13 (43.3)	
Lower	10 (33.3)	3 (10.0)	
mrT stage, *n* (%)			
mrT1–2	7 (23.3)	6 (20.0)	0.754
mrT3–4	23 (76.7)	24 (80.0)	
mrN stage, *n* (%)			0.301
mrN0	14 (46.7)	18 (60.0)	
mrN1–2	16 (53.3)	12 (40.0)	
mrEMVI, *n* (%)			0.176
Positive	8 (26.7)	13 (43.3)	
Negative	22 (73.3)	17 (56.7)	
MRF, *n* (%)			0.01
Positive	2 (6.7)	10 (33.3)	
Negative	28 (93.3)	20 (66.7)	

### 3.2 Evaluation of different parameters of TB grade

**Figure 3:**
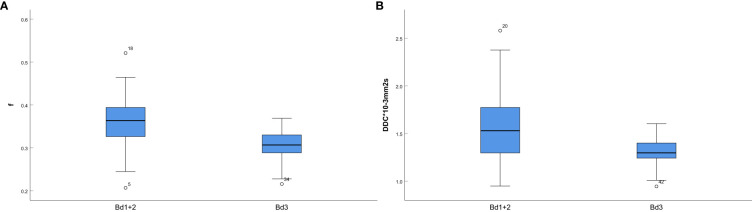
**(A)** Box-and-whisker plot for the f values in Bd 1+ Bd 2 and Bd 3; **(B)** Box-and-whisker plot for the DDC values in Bd 1+ Bd 2 and Bd 3.

The mathematical parameters obtained in this study included ADC, DDC, *D*, D*, *f*, and α, among which the DDC showed good interobserver agreement (ICC > 0.75). There were significant differences in *f* value (*p* = 0.001), DDC value (*p* < 0.001), age (*p* = 0.002), location (*p* = 0.047), and MRF (*p* = 0.01) between the two groups (details are shown in **Table 3**). The DDC value of Bd 3 was lower than that of Bd 1 + Bd 2, and the *f* value of Bd 3 was lower than that of Bd 1 + Bd 2; the difference between the two sets of mathematical parameters is presented in **Figure 3**. TB grade 3 lesions were predominantly located at the upper and middle level of the rectum. Tumors that invaded the peritoneum or were MRF-positive were associated with a higher TB grade. In addition, the overall age of patients with Bd 3 grade was lower than that of patients with Bd 1 + Bd 2 grade.

**Table 3:** Differences in parameters between the low-intermediate- and high-grade budding groups.

Parameters	Low-intermediate grade	High grade	*P*
ADC (×10^−3^ mm^2^/s)	1.210 ± 0.026	1.163 ± 0.016	0.138
*D* (×10^−3^ mm^2^/s)	0.813 (0.732, 0.848)	0.799 ± 0.0141	0.824
*D* ^*^ (×10^−3^ mm^2^/s)	12.078 (8.222, 18.167)	10.505 (8.495, 15.236)	0.745
*f* (×10^−1^ mm^2^/s)	3.584 ± 0.120	3.100 ± 0.069	0.001
DDC (×10^−3^ mm^2^/s)	1.576 ± 0.065	1.306 ± 0.027	<0.001
α (×10^−1^)	7.213 (7.051, 7.591)	7.465 ± 0.097	0.069

### 3.3 Diagnostic performance assessment

**Figure 4:**
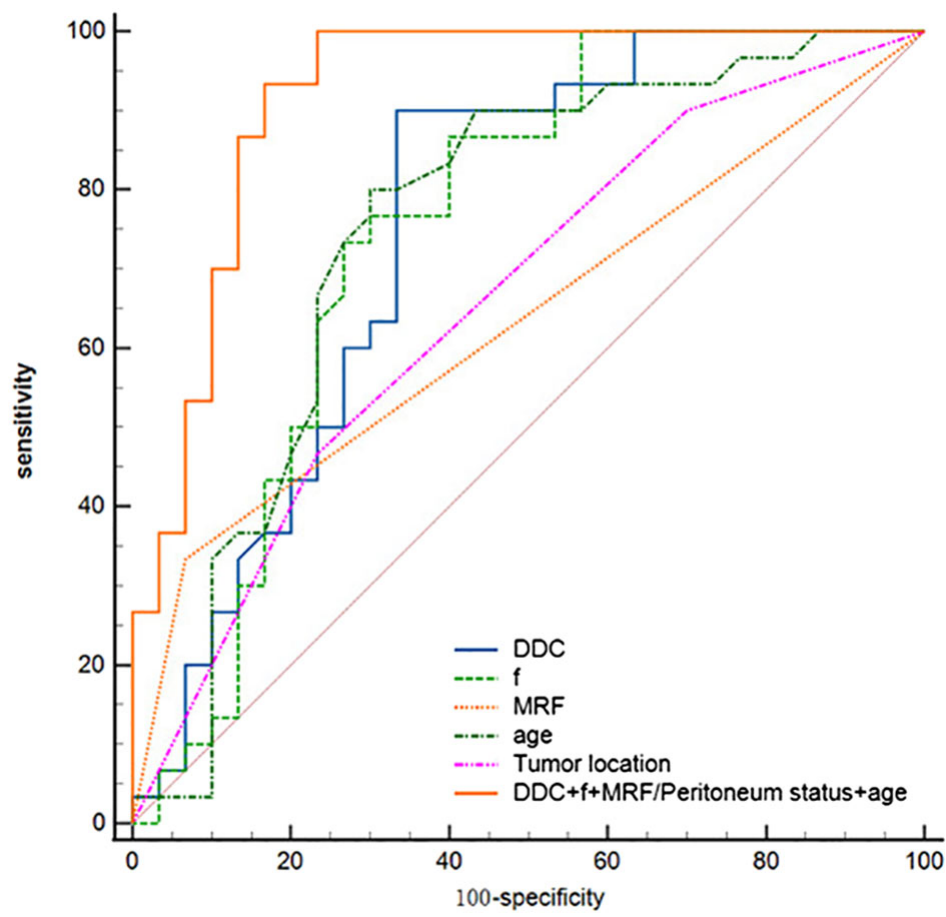
ROC curves for DDC, *f*, age, tumor location, MRF/peritoneum status, and the combined model for assessment of TB.

**Figure 5:**
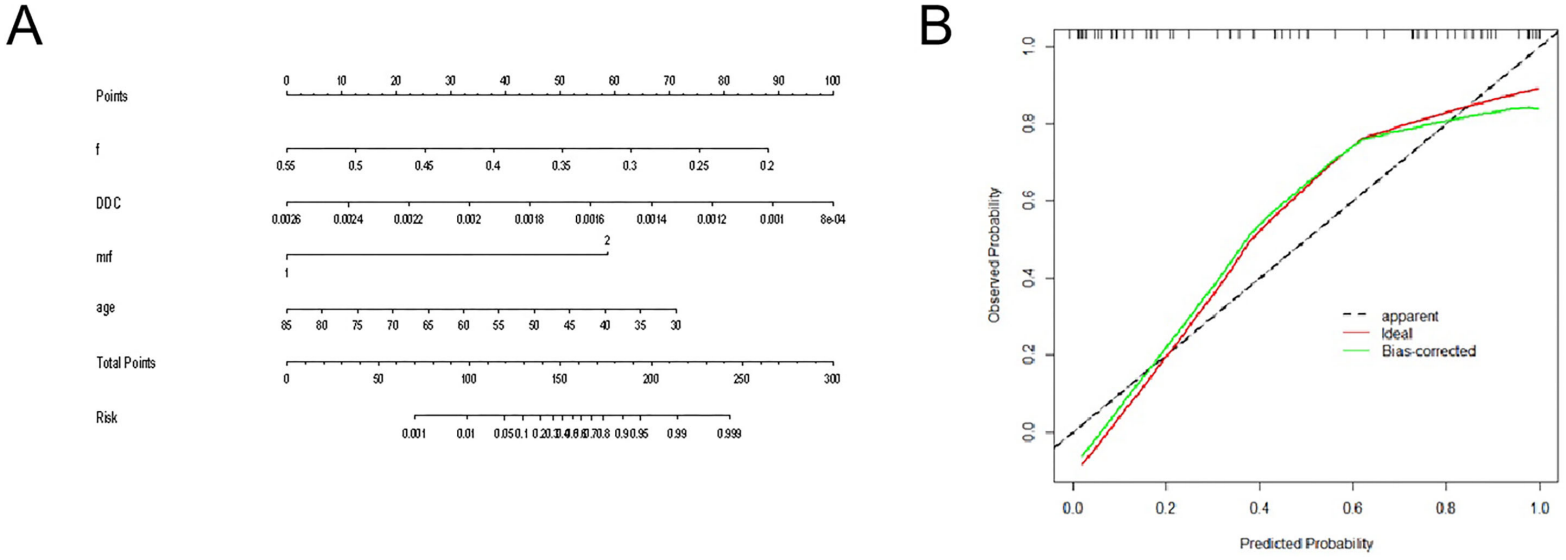
**(A)** The nomogram for predicting TB grade; **(B)** the nomogram calibration curves.

The diagnostic energy efficiencies of the *f* value (*p* = 0.001), DDC value (*p* < 0.001), age (*p* = 0.002), location (*p* = 0.047), and MRF (*p* = 0.01) are shown in **Table 4**. The diagnostic energy efficiency of each single parameter was not high. In binary logistic regression analysis, DDC, *f*, MRF, and age were identified as independent risk factors for Bd (OR < 0.001, OR < 0.001, OR = 68.012, OR = 0.895 and *p* = 0.013, *p* = 0.02, *p* = 0.031, and *p* = 0.024, respectively), as shown in **Table 5**. The AUC of the prediction model established by these indexes was 0.920 (95% CI, 0.820–0.974), with a sensitivity and specificity of 1.000 and 0.767, respectively (**Table 4**). The Delong test showed that there were significant differences in AUC between the combined model and the DDC, *f*, MRF, location, and age of individuals (*p* < 0.05). **Figure 4** presents the ROC curves of the individual variables versus the joint model. Finally, a nomogram generated to visualize the combined model and the nomogram calibration curves is shown in **Figure 5**.

**Table 4:** Diagnostic performance in the diagnosis of TB of RC.

Parameters	AUC (95% CI)	Sensitivity	Specificity	Cutoff value
DDC	0.755 (0.627–0.857)	0.900	0.667	>0.426
*f*	0.753 (0.624–0.855)	0.867	0.600	>0.373
Tumor location	0.667 (0.534–0.784)	0.900	0.333	–
MRF/Peritoneum status	0.633 (0.499–0.754)	0.333	0.933	–
Age	0.753 (0.625–0.856)	0.800	0.700	>0.449
DDC + *f* + MRF/peritoneum status + age	0.920 (0.820–0.960)	1.000	0.767	–

**Table 5:** Parameters included in the binary logistic regression analysis.

Parameters	*P*	95% CI	OR
DDC	0.014	0.000–0.000	0.000
*f*	0.021	0.000–0.051	0.000
Age	0.024	0.813–0.985	0.895
Tumor location	0.280	0.202–1.589	0.567
MRF/Peritoneum status	0.032	1.452–3,117.104	67.279

## 4 Discussion

In our study, the set of IVIM parameters based on ME, BE, and SE models was used to diagnose TB grade. Overall, our results revealed that the *f* value from the BE model, the DDC value from the SE model, MRF, and age all showed significant differences between the high-grade and low-intermediate-grade budding groups. Further investigation revealed that the joint diagnosing model including DDC, *f*, MRF, and age showed a good performance in evaluating the grading of TB.

TB is defined as a single cell or a cluster of up to four cells scattered in the interstitium at the leading edge of the invasive side of colorectal cancer and has been suggested to be related to epithelial–mesenchymal transition. Several studies have shown that TB was related to the histological grade, nerve invasion, lymph node metastasis, and tumor stage of colorectal cancer (6). IVIM can distinguish the diffusion effect of water molecules in biological tissues from the microcirculation perfusion effect. Some studies have shown that IVIM can diagnose the histological grade, TNM stage, and tumor deposition of RC (19, 20). Therefore, we assumed that IVIM may also reflect the difference between TB grades.

Our investigation further found that DDC and *f* showed significant differences between the low-intermediate and high budding grade groups. Compared with the low-intermediate budding grade group, the high budding grade group had lower DDC and *f* values. The DDC value obtained from the SE model reflects the dispersion of water molecules when ADC values are continuously distributed. This parameter also shows a certain correlation with the tissue ADC value and reflects the diffusion rate in the average voxel. Previous studies (22) have shown that the diffusion of tumor tissues may reflect their heterogeneity and could thus indirectly reflect tumor malignancy. The higher the malignancy and heterogeneity of the tumor, the more limited the diffusion of water molecules in the tumor tissue. Tumors with high budding grades had lower DDC values, indicating that the tumor had more malignant and aggressive behavior and showed greater invasion to the surrounding tissues, which is consistent with the high number of buds in the invading edge of the tumors. This explains our finding that the higher the budding grade, the lower the DDC value. The *f* value further reflects the weight of slow ADC and fast ADC, i.e., the proportion of capillary volume per unit volume to the whole volume. The results of this study showed that the high budding grade tended to have a lower *f* value. One possible reason for this may be that the *f* value is sensitive to functionalized luminal vessels. In Bd 3 rectal tumors, there may be too many tumor cells in the stroma, while the blood vessels are mostly immature and tortuous, which reduces the proportion of functional luminal vessels (23). This may explain why tumors with higher budding grades had lower *f* values.

We further identified statistical differences in age and MRF between the two budding groups in this study. Specifically, TB grade was negatively correlated with age, meaning that younger patients tended to have a higher budding grade. In recent years, the incidence of colorectal cancers diagnosed in young and middle-aged people has increased (24), indicating early onset and concealment. Most lesions in such patients show higher invasion, poor histological differentiation, wide tumor infiltration, higher TNM stage, and poor prognosis (25). The results of this study may explain the reason for this phenomenon to a certain extent; i.e., the TB grade of younger patients tends to be higher, meaning that the tumor is more invasive and prone to invasion and metastasis. MRF-positive tumors tend to have a higher TB grade, indicating that the tumor extends beyond the intestinal wall and invades the extramural mesorectal fascia, which, to some extent, reflects the invasiveness of the tumor. Most of these tumors tend to have a larger tumor invasion surface, which can increase the probability of TB.

After multivariate logistic regression analysis, age, MRF, DDC, and *f* were statistically significantly different. The AUC of the combined model established by them had a better result than those of the single DDC, *f*, MRF, location, and age, respectively. Prior studies have further analyzed the correlation between MR parameters and TB. For example, Chen et al. (26) found that the B threshold map based on DWI was correlated with TB grade. In contrast, this study explored the difference between budding grade from two independent aspects of water molecule diffusion and perfusion through the use of multiple *b* value mathematical models, more than one DWI sequence. The occurrence and development of TB were further analyzed from the perspective of water molecular perfusion. The radiomics model constructed by Peng et al. was also found to be effective at pre-operatively evaluating the TB grade in patients with cancer (27). Compared with radiomics, IVIM can better explain the occurrence and development of TB from the viewpoint of the underlying pathological mechanisms, while requiring a smaller sample size. Furthermore, compared with the above two studies, we found that age and MRF were also two independent risk factors affecting budding grade. The higher the TB grade of young patients or MRF-positive patients, the worse the prognosis. We drew the ROI of IVIM outward to the leading edge of the tumor infiltrating side, because, according to the pathological definition of TB, it mostly occurs at the leading edge of tumor invasion. Therefore, if the ROI only selects the whole tumor area, the high proportion of non-budding tumor features may reduce the difference between budding features and thus decrease the diagnostic energy efficiency.

This study has some limitations that should be mentioned: firstly, the retrospective study design would have introduced bias in patient selection. Secondly, there is currently no unified standard for the choice of *b* value of IVIM sequence. Thirdly, the number of cases was small, and large-sample and multicenter studies are therefore needed to verify the results. Finally, because of the patients who underwent neoadjuvant treatment were not included in this study, the results of this study may not be suitable for those patients.

## 5 Conclusion

In conclusion, the results of this study show that DDC and *f* value can be used to preoperatively diagnose a patient’s budding grade to a certain extent, can assist in the clinical diagnosis of the noninvasive prediction of Bd grade preoperatively, and provide more valuable information for the evaluation of tumor prognosis and formulation of targeted treatment plans. Multiple IVIM mathematical model parameters can evaluate RC budding grade preoperatively, and the joint model established by age, MRF, DDC, and *f* has better diagnostic ability for TB grade.

## Data Availability

The raw data supporting the conclusions of this article will be made available by the authors, without undue reservation.
